# 3-(2-Chloro­phen­yl)-4-hy­droxy­furan-2(5*H*)-one

**DOI:** 10.1107/S1600536811048641

**Published:** 2011-11-25

**Authors:** Zhu-Ping Xiao, Li-Cheng Yi, Jia-Liang Li, Kai-Shuang Xiang, Bo Zhang

**Affiliations:** a The Key Laboratory of Hunan Forest Products & Chemical Industry Engineering of Hunan Province and College of Chemistry & Chemical Engineering, Jishou University, Jishou 416000, People’s Republic of China

## Abstract

In the title mol­ecule, C_10_H_7_ClO_3_, the butyrolactone core, a furan-2(5*H*)-one, forms a dihedral angle of 59.21 (5)° with the benzene ring. In the crystal, two types of hydrogen bonds (O—H⋯O and C—H⋯Cl) link mol­ecules into infinite chains along the *b* axis. π–π contacts [centroid–centroid distances = 3.6359 (10) and 3.8776 (11) Å] link the chains into a three-dimensional network.

## Related literature

For the anti­bacterial activity of furan­ones, see: Xiao *et al.* (2011 ▶). For related structures, see: Peng *et al.* (2011 ▶); Xiao *et al.* (2010 ▶).
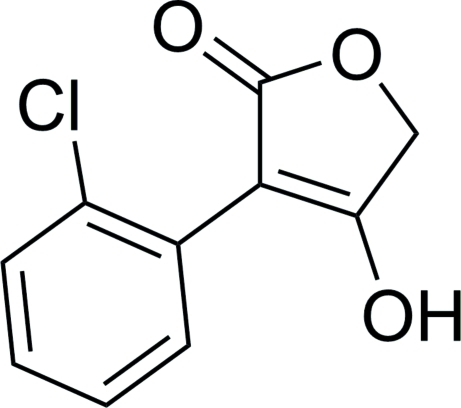


## Experimental

### 

#### Crystal data

C_10_H_7_ClO_3_
*M* *_r_* = 210.61
Monoclinic, 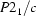

*a* = 9.9699 (15) Å
*b* = 11.8308 (18) Å
*c* = 8.1562 (12) Å
β = 104.898 (2)°
*V* = 929.7 (2) Å^3^
*Z* = 4
Mo *K*α radiation
μ = 0.39 mm^−1^
*T* = 296 K
0.30 × 0.20 × 0.20 mm

#### Data collection

Bruker SMART APEX CCD diffractometer
Absorption correction: multi-scan (*SADABS*; Sheldrick, 1996 ▶) *T*_min_ = 0.893, *T*_max_ = 0.927
7259 measured reflections
2240 independent reflections
2037 reflections with *I* > 2σ(*I*)
*R*_int_ = 0.018

#### Refinement

*R*[*F* ^2^ > 2σ(*F* ^2^)] = 0.034
*wR*(*F* ^2^) = 0.108
*S* = 1.12
2240 reflections
132 parameters
H atoms treated by a mixture of independent and constrained refinement
Δρ_max_ = 0.32 e Å^−3^
Δρ_min_ = −0.33 e Å^−3^

### 

Data collection: *SMART* (Bruker, 2007 ▶); cell refinement: *SAINT* (Bruker, 2007 ▶); data reduction: *SAINT*; program(s) used to solve structure: *SHELXS97* (Sheldrick, 2008 ▶); program(s) used to refine structure: *SHELXL97* (Sheldrick, 2008 ▶); molecular graphics: *SHELXTL* (Sheldrick, 2008 ▶); software used to prepare material for publication: *SHELXL97*.

## Supplementary Material

Crystal structure: contains datablock(s) global, I. DOI: 10.1107/S1600536811048641/pv2483sup1.cif

Structure factors: contains datablock(s) I. DOI: 10.1107/S1600536811048641/pv2483Isup2.hkl

Supplementary material file. DOI: 10.1107/S1600536811048641/pv2483Isup3.cml

Additional supplementary materials:  crystallographic information; 3D view; checkCIF report

## Figures and Tables

**Table 1:** Hydrogen-bond geometry (Å, °)

*D*—H⋯*A*	*D*—H	H⋯*A*	*D*⋯*A*	*D*—H⋯*A*
O3—H3⋯O1^i^	0.82 (3)	1.80 (3)	2.6182 (16)	170 (3)
C9—H9*B*⋯Cl1^i^	0.97	2.77	3.7126 (16)	165

Symmetry code: (i) 
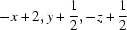
.

**Fig. 1.**
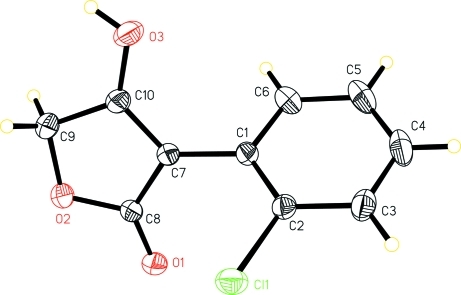
Molecular structure of the title compound, showing the atom-numbering scheme. Displacement ellipsoids are drawn at the 30% probability level.

**Fig. 2.**
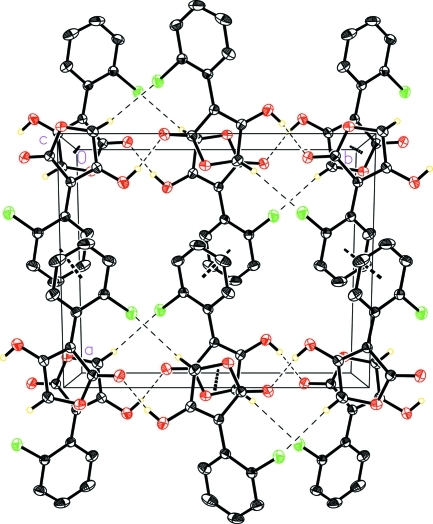
A unit cell packing diagram of the title compound showing a three-dimensional network built through intermolecular hydrogen bonds and π–π contacts.

**Fig. 3.**
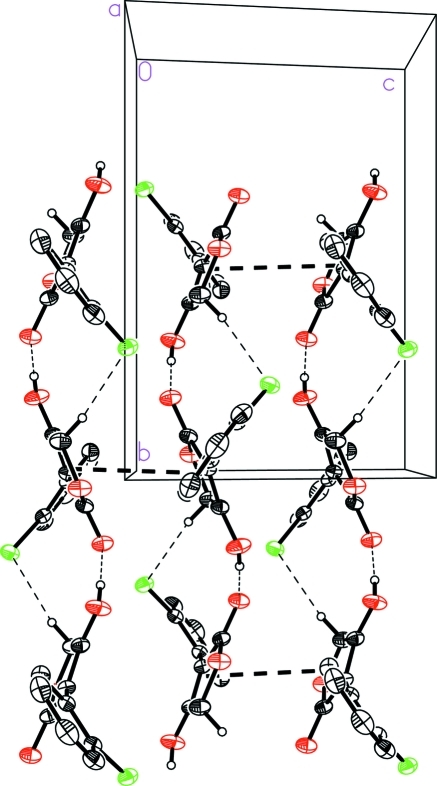
A unit cell packing diagram of the title compound

## supplementary crystallographic information

### Comment

Recently, we have reported the antibacterial activities of a few γ-butyrolactones (furanones) (Xiao *et al.*, 2011). As a part of our ongoing studies of γ-butyrolactones (Xiao *et al.*, 2010), we herein report the crystal structure of the title compound.

In the title compound (Fig. 1), the butyrolactone moiety makes a dihedral angle of 59.21 (5) ° with the benzene ring. Relatively strong intermolecular hydrogen bonds (O—H···O) link molecules into an infinite chain running along the *b* axis, which is further consolidated by weak intermolecular C—H···Cl interactions. There are π–π contacts between benzene rings and butyrolactone rings with centroid–centroid distances 3.6359 (10) and 3.8776 (11) Å, respectively (Fig. 2). The molecular dimensions in the title molecule agree very well with the corresponding molecular dimensions reported in a few similar structures (Peng *et al.*, 2011; Xiao *et al.*, 2010).

### Experimental

A dropwise solution of 2-ethoxy-2-oxoethyl 2-(2-chlorophenyl)acetate (1.03 g, 4 mmol) in dry THF was added to a suspension of NaH in dry THF in an ice cold bath. The stirring was maintained at room temperature for 6 h. Water was added and the solution was extracted twice with ethyl ether. The aqueous phase was cooled to 273 K and then acidified with concentrated hydrochloric acid to give a solid precipitate. The title compound thus obtained was crystallized from ethanol-water (2:1) to give the colorless blocks suitable for single-crystal structure determination.

### Refinement

The H-atoms bonded to C-atoms were placed in geometrically idealized positions and constrained to ride on their parent atoms with C—H = 0.93 and 0.97 Å for aryl and methylene type H atoms, respectively, with *U*_iso_(H) = 1.2 *U*_eq_(C). The hydroxyl H atom was located from a difference Fourier map and was allowed to refine freely.

### Crystal data

**Table:**

C_10_H_7_ClO_3_	*F*(000) = 432
*M**_r_* = 210.61	*D*_x_ = 1.505 Mg m^−^^3^
Monoclinic, *P*2_1_/*c*	Mo *K*α radiation, λ = 0.71073 Å
Hall symbol: -P 2ybc	Cell parameters from 2125 reflections
*a* = 9.9699 (15) Å	θ = 2.7–27.8°
*b* = 11.8308 (18) Å	µ = 0.39 mm^−^^1^
*c* = 8.1562 (12) Å	*T* = 296 K
β = 104.898 (2)°	Block, colorless
*V* = 929.7 (2) Å^3^	0.30 × 0.20 × 0.20 mm
*Z* = 4	

### Data collection

**Table:**

Bruker SMART APEX CCD diffractometer	2240 independent reflections
Radiation source: fine-focus sealed tube	2037 reflections with *I* > 2σ(*I*)
graphite	*R*_int_ = 0.018
φ and ω scan	θ_max_ = 28.0°, θ_min_ = 2.1°
Absorption correction: multi-scan (*SADABS*; Sheldrick, 1996)	*h* = −13→10
*T*_min_ = 0.893, *T*_max_ = 0.927	*k* = −15→15
7259 measured reflections	*l* = −10→10

### Refinement

**Table:**

Refinement on *F*^2^	Secondary atom site location: difference Fourier map
Least-squares matrix: full	Hydrogen site location: inferred from neighbouring sites
*R*[*F*^2^ > 2σ(*F*^2^)] = 0.034	H atoms treated by a mixture of independent and constrained refinement
*wR*(*F*^2^) = 0.108	*w* = 1/[σ^2^(*F*_o_^2^) + (0.0607*P*)^2^ + 0.1971*P*] where *P* = (*F*_o_^2^ + 2*F*_c_^2^)/3
*S* = 1.12	(Δ/σ)_max_ = 0.001
2240 reflections	Δρ_max_ = 0.32 e Å^−^^3^
132 parameters	Δρ_min_ = −0.33 e Å^−^^3^
0 restraints	Extinction correction: *SHELXL97* (Sheldrick, 2008), Fc^*^=kFc[1+0.001xFc^2^λ^3^/sin(2θ)]^-1/4^
Primary atom site location: structure-invariant direct methods	Extinction coefficient: 0.021 (4)

### Special details

**Table:**

Geometry. All e.s.d.'s (except the e.s.d. in the dihedral angle between two l.s. planes) are estimated using the full covariance matrix. The cell e.s.d.'s are taken into account individually in the estimation of e.s.d.'s in distances, angles and torsion angles; correlations between e.s.d.'s in cell parameters are only used when they are defined by crystal symmetry. An approximate (isotropic) treatment of cell e.s.d.'s is used for estimating e.s.d.'s involving l.s. planes.
Refinement. Refinement of *F*^2^ against ALL reflections. The weighted *R*-factor *wR* and goodness of fit *S* are based on *F*^2^, conventional *R*-factors *R* are based on *F*, with *F* set to zero for negative *F*^2^. The threshold expression of *F*^2^ > σ(*F*^2^) is used only for calculating *R*-factors(gt) *etc*. and is not relevant to the choice of reflections for refinement. *R*-factors based on *F*^2^ are statistically about twice as large as those based on *F*, and *R*- factors based on ALL data will be even larger.

### Fractional atomic coordinates and isotropic or equivalent isotropic displacement parameters (Å^2^)

**Table:**

	*x*	*y*	*z*	*U*_iso_*/*U*_eq_	
C1	0.70093 (13)	0.46964 (11)	0.22948 (16)	0.0334 (3)	
C2	0.62894 (13)	0.38058 (11)	0.13494 (17)	0.0350 (3)	
C3	0.48970 (15)	0.36149 (14)	0.1245 (2)	0.0475 (4)	
H3A	0.4435	0.3010	0.0617	0.057*	
C4	0.42063 (16)	0.43319 (17)	0.2082 (2)	0.0567 (4)	
H4	0.3274	0.4206	0.2022	0.068*	
C5	0.48850 (17)	0.52330 (17)	0.3006 (2)	0.0556 (4)	
H5	0.4408	0.5719	0.3553	0.067*	
C6	0.62747 (16)	0.54126 (14)	0.3117 (2)	0.0455 (3)	
H6	0.6729	0.6019	0.3748	0.055*	
C7	0.84833 (13)	0.49225 (10)	0.23989 (17)	0.0337 (3)	
C8	0.96157 (13)	0.41569 (11)	0.31055 (18)	0.0365 (3)	
C9	1.05960 (15)	0.57272 (12)	0.2315 (2)	0.0459 (3)	
H9A	1.0901	0.5790	0.1281	0.055*	
H9B	1.1076	0.6290	0.3119	0.055*	
C10	0.90647 (14)	0.58691 (11)	0.19598 (18)	0.0380 (3)	
Cl1	0.71082 (4)	0.29321 (3)	0.01933 (5)	0.04628 (15)	
H3	0.897 (3)	0.730 (2)	0.125 (3)	0.080 (7)*	
O1	0.95949 (11)	0.32284 (8)	0.37570 (15)	0.0465 (3)	
O2	1.08432 (10)	0.46088 (9)	0.30186 (16)	0.0482 (3)	
O3	0.84132 (12)	0.67963 (10)	0.12899 (18)	0.0564 (3)	

### Atomic displacement parameters (Å^2^)

**Table:**

	*U*^11^	*U*^22^	*U*^33^	*U*^12^	*U*^13^	*U*^23^
C1	0.0298 (6)	0.0325 (6)	0.0387 (6)	0.0021 (4)	0.0102 (5)	0.0046 (5)
C2	0.0319 (6)	0.0325 (6)	0.0400 (6)	0.0003 (5)	0.0082 (5)	0.0070 (5)
C3	0.0328 (7)	0.0490 (8)	0.0568 (9)	−0.0059 (6)	0.0046 (6)	0.0136 (7)
C4	0.0296 (7)	0.0776 (12)	0.0647 (10)	0.0058 (7)	0.0156 (7)	0.0213 (9)
C5	0.0430 (8)	0.0773 (12)	0.0514 (9)	0.0217 (8)	0.0208 (7)	0.0107 (8)
C6	0.0417 (7)	0.0505 (8)	0.0454 (7)	0.0105 (6)	0.0128 (6)	−0.0012 (6)
C7	0.0306 (6)	0.0290 (6)	0.0419 (6)	−0.0011 (4)	0.0101 (5)	−0.0033 (5)
C8	0.0318 (6)	0.0314 (6)	0.0470 (7)	0.0004 (5)	0.0117 (5)	−0.0048 (5)
C9	0.0356 (7)	0.0384 (7)	0.0648 (9)	−0.0071 (5)	0.0149 (6)	−0.0014 (6)
C10	0.0345 (6)	0.0311 (6)	0.0469 (7)	−0.0040 (5)	0.0080 (5)	−0.0031 (5)
Cl1	0.0483 (2)	0.0362 (2)	0.0521 (2)	0.00220 (13)	0.00863 (16)	−0.00722 (13)
O1	0.0408 (5)	0.0334 (5)	0.0656 (7)	0.0049 (4)	0.0140 (5)	0.0054 (4)
O2	0.0304 (5)	0.0397 (5)	0.0752 (7)	0.0007 (4)	0.0146 (5)	0.0014 (5)
O3	0.0410 (6)	0.0344 (5)	0.0876 (9)	−0.0053 (4)	0.0056 (6)	0.0141 (6)

### Geometric parameters (Å, °)

**Table:**

C1—C2	1.3912 (18)	C6—H6	0.9300
C1—C6	1.3987 (19)	C7—C10	1.3510 (18)
C1—C7	1.4745 (17)	C7—C8	1.4465 (17)
C2—C3	1.3873 (19)	C8—O1	1.2228 (17)
C2—Cl1	1.7377 (14)	C8—O2	1.3539 (16)
C3—C4	1.379 (3)	C9—O2	1.4382 (18)
C3—H3A	0.9300	C9—C10	1.488 (2)
C4—C5	1.378 (3)	C9—H9A	0.9700
C4—H4	0.9300	C9—H9B	0.9700
C5—C6	1.381 (2)	C10—O3	1.3195 (17)
C5—H5	0.9300	O3—H3	0.82 (3)
			
C2—C1—C6	117.73 (12)	C10—C7—C8	106.29 (11)
C2—C1—C7	122.26 (11)	C10—C7—C1	128.63 (12)
C6—C1—C7	119.97 (12)	C8—C7—C1	125.00 (11)
C3—C2—C1	121.53 (13)	O1—C8—O2	119.61 (12)
C3—C2—Cl1	118.17 (11)	O1—C8—C7	129.56 (12)
C1—C2—Cl1	120.26 (10)	O2—C8—C7	110.81 (11)
C4—C3—C2	119.25 (15)	O2—C9—C10	104.08 (11)
C4—C3—H3A	120.4	O2—C9—H9A	110.9
C2—C3—H3A	120.4	C10—C9—H9A	110.9
C5—C4—C3	120.60 (14)	O2—C9—H9B	110.9
C5—C4—H4	119.7	C10—C9—H9B	110.9
C3—C4—H4	119.7	H9A—C9—H9B	109.0
C4—C5—C6	119.86 (15)	O3—C10—C7	126.86 (13)
C4—C5—H5	120.1	O3—C10—C9	123.04 (12)
C6—C5—H5	120.1	C7—C10—C9	110.09 (12)
C5—C6—C1	121.02 (16)	C8—O2—C9	108.65 (10)
C5—C6—H6	119.5	C10—O3—H3	111.0 (18)
C1—C6—H6	119.5		
			
C6—C1—C2—C3	−1.21 (19)	C6—C1—C7—C8	119.84 (15)
C7—C1—C2—C3	−179.04 (12)	C10—C7—C8—O1	175.41 (15)
C6—C1—C2—Cl1	176.21 (10)	C1—C7—C8—O1	−1.6 (2)
C7—C1—C2—Cl1	−1.61 (17)	C10—C7—C8—O2	−2.92 (16)
C1—C2—C3—C4	0.7 (2)	C1—C7—C8—O2	−179.92 (12)
Cl1—C2—C3—C4	−176.74 (12)	C8—C7—C10—O3	−178.86 (15)
C2—C3—C4—C5	0.4 (2)	C1—C7—C10—O3	−2.0 (2)
C3—C4—C5—C6	−0.9 (3)	C8—C7—C10—C9	2.03 (16)
C4—C5—C6—C1	0.4 (2)	C1—C7—C10—C9	178.90 (13)
C2—C1—C6—C5	0.6 (2)	O2—C9—C10—O3	−179.72 (14)
C7—C1—C6—C5	178.52 (14)	O2—C9—C10—C7	−0.57 (17)
C2—C1—C7—C10	121.29 (16)	O1—C8—O2—C9	−175.94 (13)
C6—C1—C7—C10	−56.5 (2)	C7—C8—O2—C9	2.58 (16)
C2—C1—C7—C8	−62.39 (19)	C10—C9—O2—C8	−1.25 (16)

### Hydrogen-bond geometry (Å, °)

**Table:**

*D*—H···*A*	*D*—H	H···*A*	*D*···*A*	*D*—H···*A*
O3—H3···O1^i^	0.82 (3)	1.80 (3)	2.6182 (16)	170 (3)
C9—H9B···Cl1^i^	0.97	2.77	3.7126 (16)	165.

Symmetry codes: (i) −*x*+2, *y*+1/2, −*z*+1/2.
